# Prognostic Significance of CD276 in Non-small Cell Lung Cancer

**DOI:** 10.1515/med-2019-0076

**Published:** 2019-11-07

**Authors:** Changgong Zhang, Xuezhi Hao

**Affiliations:** 1 Department of Oncology, National Cancer Center/National Clinical Research Center for Cancer/ Cancer Hospital, Chinese Academy of Medical Sciences and Peking Union Medical College, Beijing, 100021, China

**Keywords:** Non-small cell lung cancer, CD276, Oncomine, Prognosis

## Abstract

**Background:**

The expression and significance of CD276 in non-small cell lung cancer (NSCLC) was explored.

**Method:**

The BioGPS database was used to analyze the expression level of CD276 in normal tissues. Studies on the expression of CD276 in NSCLC patients using the Oncomine database. The prognostic roles of CD276 in NSCLC was studied using the Kaplan-Meier plotter database.

**Result:**

The BioGPS database showed CD276 expression in all the human normal tissues. Compared with normal lung tissue, CD276 gene highly expressed in NSCLC tissue at mRNA level (P<0.05). The expression level of CD276 gene was negatively correlated with overall survival (OS) of NSCLC patients. Subgroup analysis showed that CD276 expression level had a significant effect on OS of patients with lung adenocarcinoma, while in squamous cell carcinoma its expression level had no significant effect on OS.

**Conclusion:**

According to the information mined from the tumor gene database, CD276 mRNA was found highly expressed in NSCLC tissue and the expression of CD276 has a significant impact on survival of NSCLC patients, which provides an important theoretical basis for further study of the role of CD276 in the occurrence and development of NSCLC.

## 1 Introduction

Lung cancer has become the leading cause of cancer-related deaths worldwide. Its incidence and mortality are increasing year by year [1]. Non-small cell lung cancer (NSCLC) accounts for approximately 80% of all lung cancer patients, and more than half of NSCLC patients are locally advanced or distantly metastatic at the time of diagnosis [2]. Therefore, improving the early diagnosis rate of NSCLC, discovering new indicators for evaluating prognosis, and new targets for anti-tumor therapy have become the focus of current NSCLC research.

CD276 is a type I transmembrane protein and its extracellular domain consists of two immunoglobulin constant (IgC) and variable (IgV) domains [3]. CD276 is a co-stimulatory/co-inhibitory molecule. When Chapoval et al first reported CD276, it was found to have a co-stimulatory effect on CD4 ^+^ and CD8 ^+^ cells. As a co-stimulatory molecule, CD276 signal induces cellular immunity. CD276 enhances the induction of cytotoxic T cells and selectively stimulates interferon gamma production (IFNγ) [4]. However, with the deepening of research, experiments have shown that the co-inhibition of CD276 in humans and mice can inhibit Treg cells [5], thereby allowing tumors to escape immune responses, and the mechanism may be related to NFAT, NF-kB and AP-1 factors [6]. Taken together, these results suggest that the immune function of CD276 remains controversial, with conflicting co-stimulatory and co-suppressive functions, which may be associated with CD276 and multiple possible associated molecular expressions, such as programmed death receptor 1 (PD-1), cytotoxic T lymphocyte-associated antigen 4 (CTLA-4), or related to the presence of different types of immune response-related cells.

There are few studies on whether CD276 plays a role in NSCLC and its function in the development of NSCLC. This study used a database to analyze the expression of CD276 gene in NSCLC and its impact on prognosis.

## 2 Materials and methods

### 2.1 The BioGPS database

Analysis of CD276 expression in normal human tissues was made using BioGPS database [7].

### 2.2 The Oncomine database

Oncomine database is currently the largest number of oncogene arrays integrated data mining platform in the world [8]. In this database, the conditions for filtering and data mining can be set. The screening conditions set in this study are: “Gene: CD276”; “Analysis Type: Lung Cancer vs. Normal Analysis”; “Cancer Type: Lung Cancer”; “Sample Type: Clinical Specimen”; “Data Type: mRNA”.

### 2.3 The Kaplan-Meier Plotter

Online survival analysis was performed using the lung cancer dataset of the Kaplan-Meier Plotter (KM Plotter) [9]. The screening conditions are as follows: “Cancer: Lung Cancer”; “Gene: CD276”; “Survival: OS”.

### 2.4 The String database explores protein-protein interactions

The database is a search tool for analyzing biological gene or protein interactions, including biological databases and network resources for proven and predictable protein-protein interactions [10]. In this study, the search term used was “CD276”, with the species type as “Homo sapiens”, the confidence level at “Medium 0.400”, and the maximum number of interactions as 10.

### 2.5 Statistical processing

The difference in CD276 gene expression between NSCLC and normal lung tissue was analyzed by *t* test. The relationship between CD276 gene expression and prognosis was analyzed by Kaplan-Meier model. The survival rate between the two groups was compared by Log-Rank test. The test level was α=0.05. The data used were analyzed by online statistical analysis. P<0.05 was considered statistically significant.

## 3 Results

### 3.1 Expression of CD276 in normal human tissues

**Figure 1:**
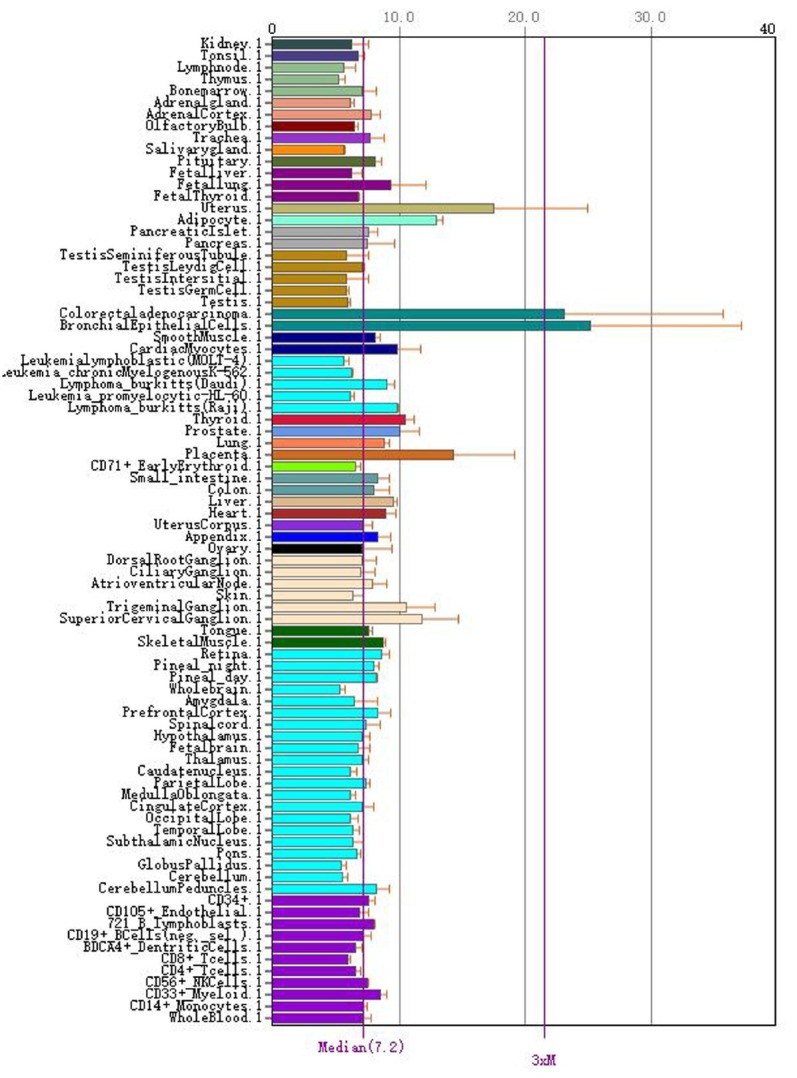
Expression of CD276 in normal human tissues

The BioGPS database showed that the CD276 gene was expressed in all normal tissues of the human body, with higher levels in bronchial epithelial cells and the uterus (Figure 1).

### 3.2 Expression of CD276 in common tumor types

**Figure 2:**
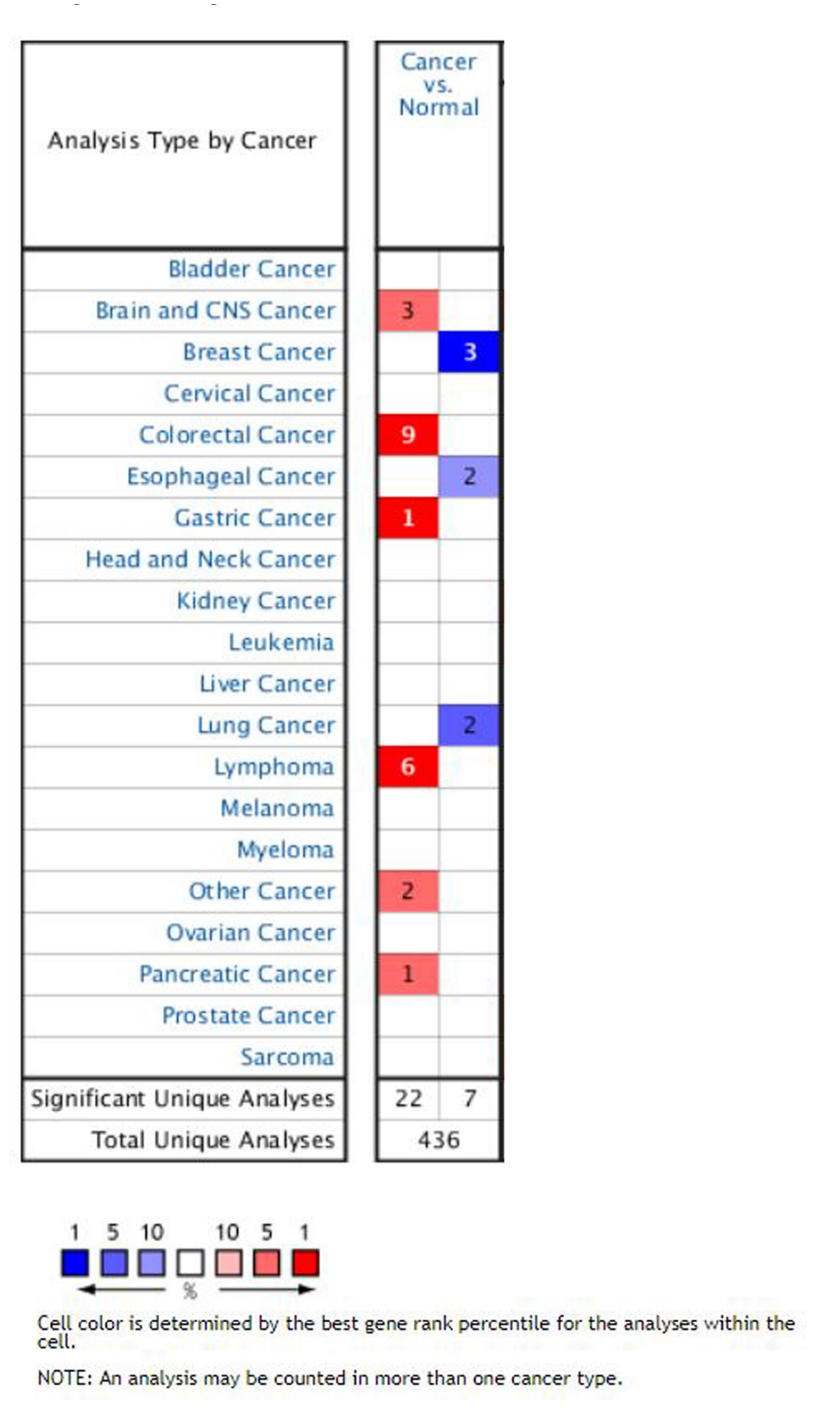
Expression of CD276 in common tumor types

A total of 436 different types of microarray studies were collected in the Oncomine database, and 29 of them had statistically significant differences in CD276 expression, including 22 studies of increased CD276 expression and three studies of decreased CD276 expression (Figure 2).

### 3.3 Expression of CD276 in NSCLC

**Figure 3:**
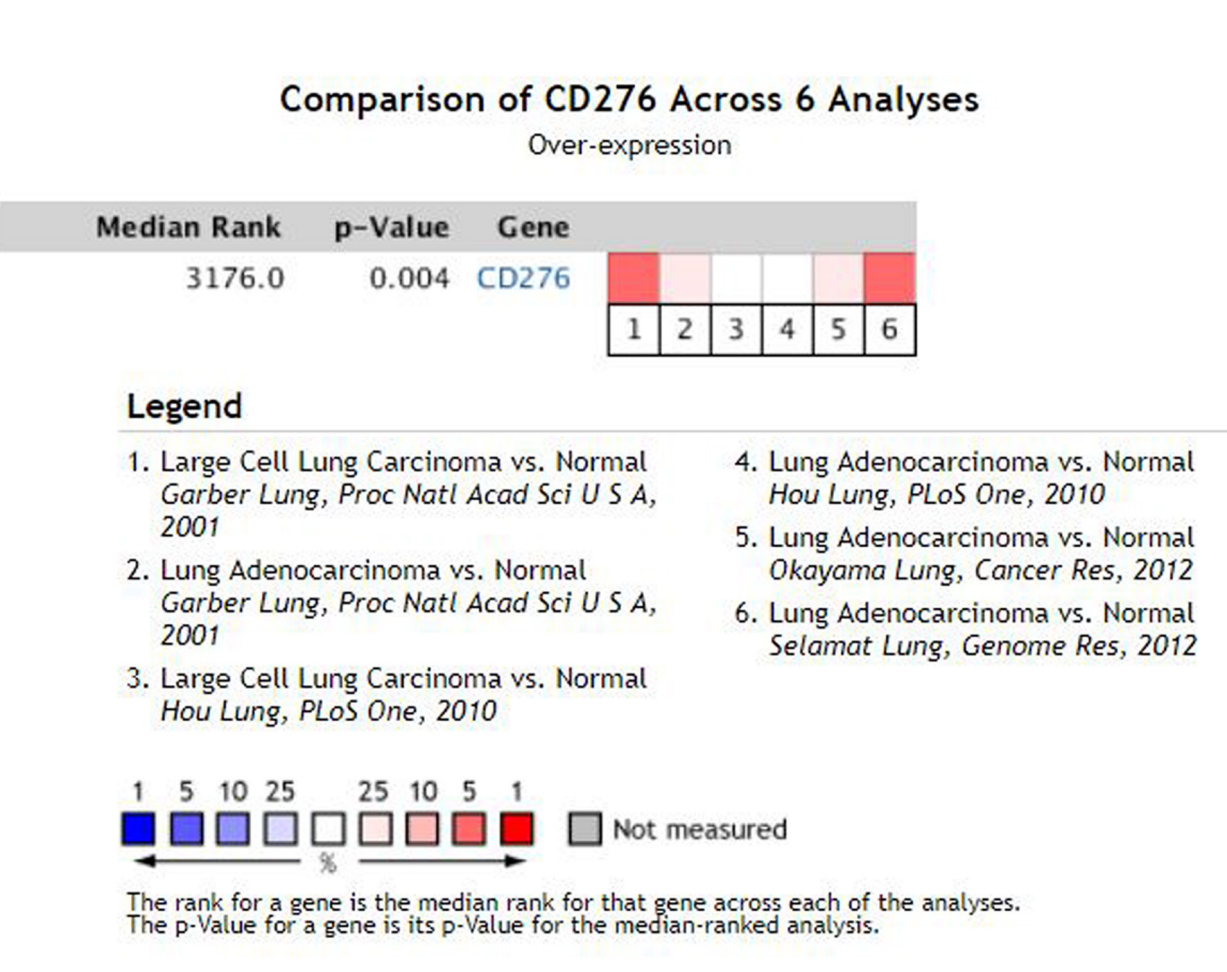
Differential expression of CD276 in NSCLC and normal lung tissues

A search in the Oncomine database found that 6 studies had focused on the expression of CD276 in NSCLC and normal lung tissue since 2003, including a total of 591 samples (Figure 3). The article was published in *Proc Natl Acad Sci* [11], *PLoS One* [12], *Cancer Res* [13], *Genome Res* [14]. A meta-analysis of the results of these 6 studies revealed that CD276 was highly expressed in NSCLC tissues compared with normal lung tissues, with a median rank of 3176 and P=0.004, indicating statistical significance.

### 3.4 Differential expression of CD276 in NSCLC and normal lung tissues

**Figure 4:**
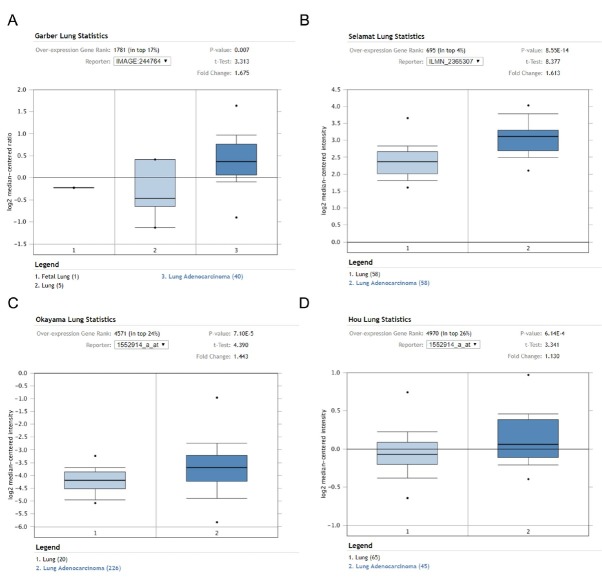
Differential expression of CD276 in NSCLC and normal lung tissues

Data mining of Oncomine showed that CD276 was highly expressed in different types of NSCLC research arrays compared with normal lung tissue, and the difference was statistically significant (Figure 4). In the study by Garber et al, CD276 mRNA expression was 1.675 times higher in lung adenocarcinoma (40 cases) than normal lung tissue (5 cases) (P = 0.007, Figure 4A). In the study by Selamat et al, the expression of CD276 mRNA was increased by 1.613 times in lung adenocarcinoma (58 cases) compared with normal lung tissue (58 cases) (P = 8.55E-14, Figure 4B). In Okayama et al, the expression of CD276 mRNA was 1.443 times higher in lung adenocarcinoma (226 cases) than in normal lung tissues (20 cases) (P = 7.10E-5, Figure 4C). In

Hou et al, the expression of CD276 mRNA was 1.13 times higher in lung adenocarcinoma (45 cases) than in normal lung tissues (65 cases) (P = 6.14E-4, Figure 4D).

### 3.5 Relationship between CD276 expression level and prognosis of patients with NSCLC

**Figure 5:**
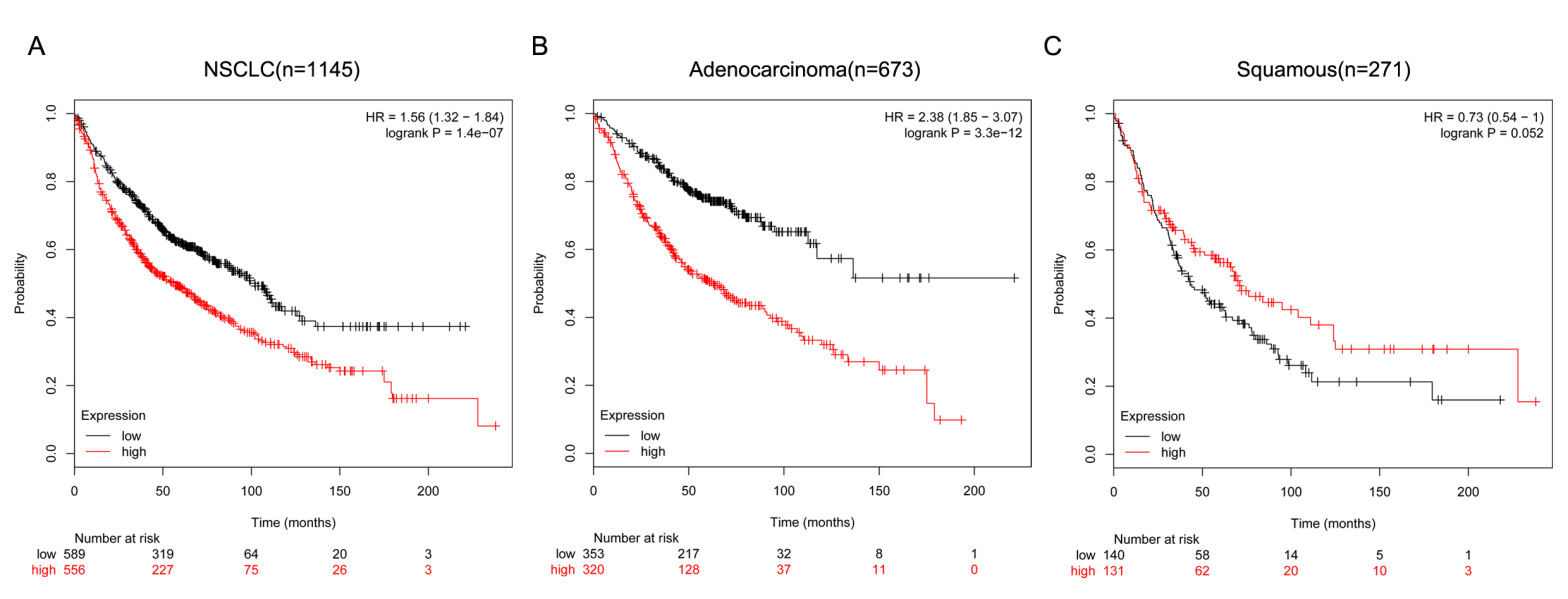
Relationship between CD276 expression level and prognosis of patients with NSCLC

To further clarify the relationship between CD276 expression level and the prognosis of patients with NSCLC, KM Plotter database analysis showed a survival curve of 1145 patients with high CD276 expression and low expression group, and found that CD276 expression level has a significant impact on overall survival of patients. Compared with 589 patients with low-expression NSCLC, 556 patients with high CD276 expression had significantly lower OS (HR=1.56, logrank P=1.4e-07, Figure 5A). This indicates that CD276 expression is a risk factor for prognosis in patients with NSCLC. Further subgroup analysis showed that in 673 patients with lung adenocarcinoma, high RPA3 expression levels had a significant effect on OS (HR=2.38, logrankP=3.3e-12, Figure 5B), whereas in 271 patients with lung squamous cell carcinoma, Its high expression level had no significant effect on OS (HR=0.73, logrankP=0.052, Figure 5C).

### 3.6 Protein network interacting with CD276

**Figure 6:**
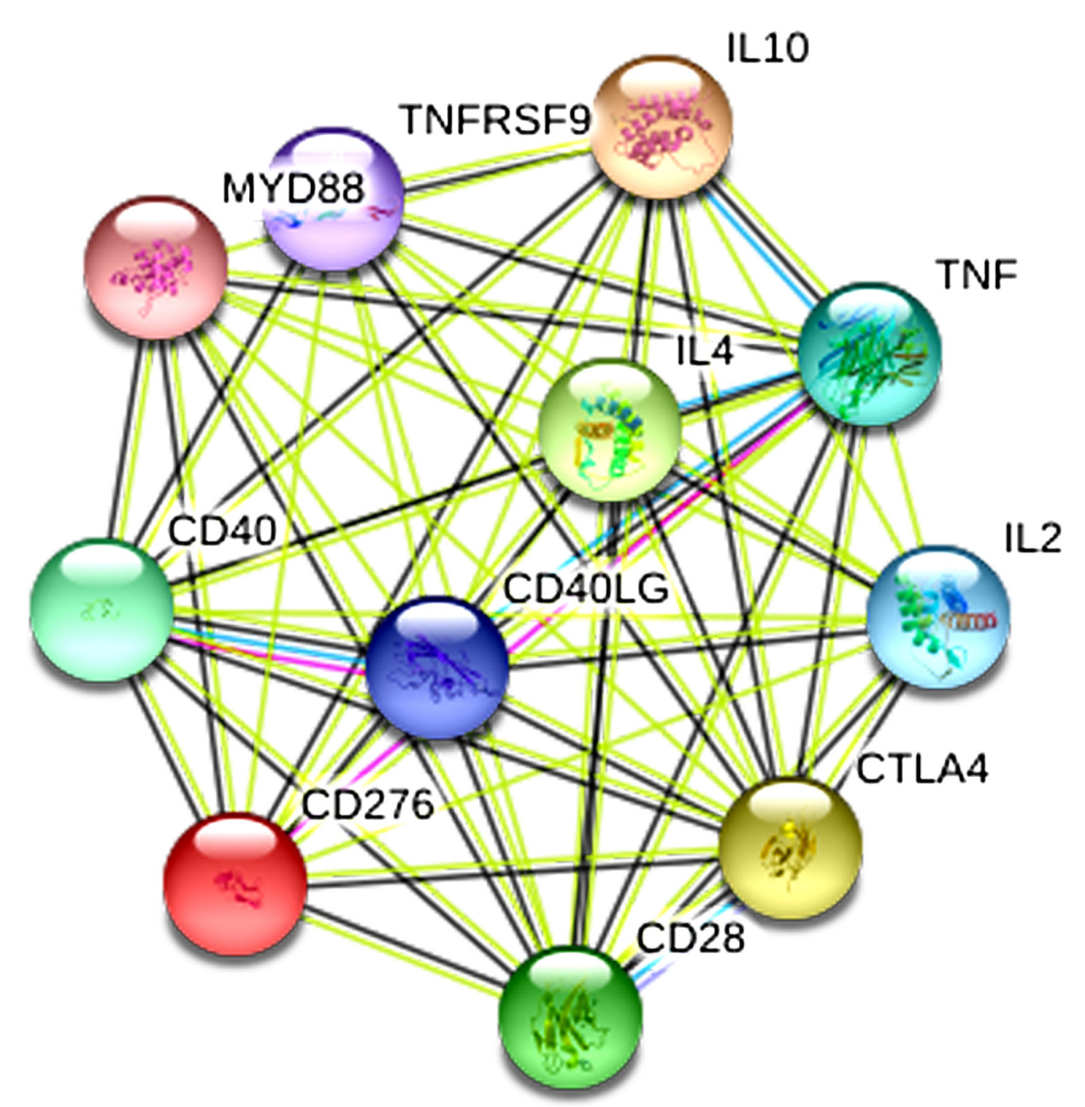
Protein network interacting with CD276

The protein network interacting with CD276 was obtained by String database analysis and the number of nodes was 11 (Figure. 6). Among them, CTLA4, IL2, IL4 interacted with CD276 in the PPI network (score > 0.900), and the main biological processes involved were regulation of immunoglobulin production, regulation of cytokine production and regulation of lymphocyte proliferation.

## 4 Discussion

At present, the incidence and mortality of lung cancer in the world are the highest among all tumors. In recent years, the application of chemotherapy, radiotherapy, targeted drugs and immunotherapy in lung cancer has significantly improved the 5-year survival rate of NSCLC [15]. Due to the problems of radiochemotherapy resistance, treatment failure, tumor recurrence and metastasis, the long-term prognosis is still very poor, and new research is urgently needed to find effective diagnostic methods and therapeutic targets.

Studies have found that CD276 is expressed at both transcriptional and translational levels in six different non-small cell lung cancer cell lines [15]. Studies have shown that CD276 helps to suppress normal immune responses and can cause cancer progression in patients with NSCLC. In histological examinations of patients with NSCLC, CD276 expression was significantly associated with NSCLC lymph node metastasis and advanced TNM stage, and CD276 was found to have an inhibitory effect on the immune system of NSCLC [16]. Another study analyzed tissue samples from 105 patients with NSCLC, not only found that high expression of CD276 protein is associated with poor prognosis, but also that CD276 can participate in the progression of NSCLC by inducing monocytes to develop into anti-inflammatory cells [17]. In another study, high CD276 expression was observed to be associated with high tumor grade and short overall survival, possibly in synergy with Treg cells, allowing tumors to evade immune responses. However, there are also statistics showing that abnormal expression of CD276 is associated with lymph node metastasis and late stage of NSCLC in TNM stage. However, there is no significant association between CD276 and overall survival in NSCLC [18, 19].

The BioGPS database is an online gene annotation database that contains information on public resources and data generated by Novartis Research [20]. The results based on BioGPS database mining showed that CD276 gene was expressed in all normal tissues of the human body, especially in bronchial epithelial cells. The Oncomine database integrates RNA-seq and DNA-seq data from the Cancer Genome Atlas (TCGA) and the Gene Expression Omnibus (GEO), and published literature to analyze differential expression in cancer and normal tissues [21]. This study used the Oncomine database to conduct a meta-analysis of lung cancer-related studies. The results showed that CD276 was highly expressed in NSCLC tissues in 591 specimens from 6 studies. The KM Plotter database is the world’s most widely accepted prognostic-related online analysis database, containing mRNA profiles of five cancers (breast, ovarian, lung, stomach, liver cancer), which can be used for relevant prognostic analysis of 54675 genes [22]. This study was the first to analyze the prognostic relationship between CD276 and NSCLC through the KM plotter data platform. The results showed that the expression level of CD276 was correlated with the OS of NSCLC, and the OS of patients with high CD276 expression was significantly decreased, indicating that high expression of CD276 is an independent risk factor for poor prognosis in patients with NSCLC. Subgroup analysis showed that the expression level of CD276 had a significant effect on the prognosis of patients with lung adenocarcinoma, while in patients with squamous cell carcinoma, the expression level had no significant effect on the prognosis. At present, the specific role of CD276 in the development of NSCLC and its molecular mechanism have not been studied.

In summary, through the deep mining of NSCLC-related gene information in the database, it was found that CD276 is highly expressed in NSCLC tissues and is associated with the prognosis of NSCLC. The results of these researches provide clues and evidence for further experimental research, and provide theoretical support for further research on molecular mechanisms.
